# 6-Nitro-2,3-dihydro-1*H*-pyrrolo­[2,1-*c*][1,4]benzodiazepine-5,11(10*H*,11a*H*)-dione

**DOI:** 10.1107/S1600536811024500

**Published:** 2011-07-09

**Authors:** Abdessamad Jebani, Hafid Zouihri, Ahmed El Hakmaoui, Saïd Lazar, Mohamed Akssira

**Affiliations:** a Laboratoire de Chimie Biorganique & Analytique, URAC 22, Université Hassan II, Mohammedia-Casablanca, BP 146, 20800 Mohammedia, Morocco; b Laboratoires de Diffraction des Rayons X, Centre National pour la Recherche Scientifique et Technique, Rabat, Morocco; c Laboratoire de Biochimie, Environnement & Agroalimentaire, URAC 36, Université Hassan II, Mohammedia-Casablanca, BP 146, 20800 Mohammedia, Morocco

## Abstract

In the two mol­ecules of the asymmetric unit of the title compound, C_12_H_11_N_3_O_4_, the seven-membered diazepine ring adopts a boat conformation (with the two phenyl­ene C atoms representing the stern and the methine C atom the prow). The five-membered pyrrole ring, which has an envelope conformation, makes dihedral angles of 60.47 (10) and 54.69 (9)° with the benzene ring of the benzodiazepine unit in the two mol­ecules. In the crystal, inter­molecular N—H⋯O hydrogen bonds and π–π stacking inter­actions [centroid–centroid distance = 3.8023 (7)–3.8946 (7) Å] lead to the formation of a three-dimensional framework.

## Related literature

For the biological activity of pyrrolo­[2,1-*c*][1,4]benzodiazepine derivatives, see: Dervan (1986 ▶); Leimgruber *et al.* (1975 ▶); Da Settimo *et al.* (2007 ▶); Herpin *et al.* (2000 ▶); Arima *et al.* (1983 ▶). 
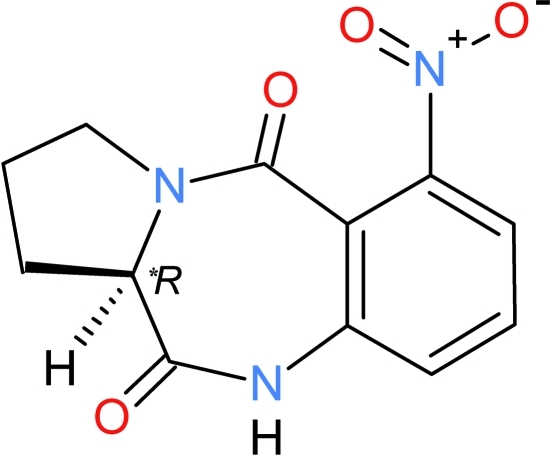


## Experimental

### 

#### Crystal data

C_12_H_11_N_3_O_4_
*M* *_r_* = 261.24
Monoclinic, 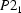

*a* = 10.7364 (2) Å
*b* = 6.8925 (1) Å
*c* = 16.3901 (3) Å
β = 101.870 (1)°
*V* = 1186.94 (4) Å^3^
*Z* = 4
Mo *K*α radiation
μ = 0.11 mm^−1^
*T* = 296 K
0.23 × 0.20 × 0.15 mm

#### Data collection

Bruker APEXII CCD detector diffractometer
14768 measured reflections
2543 independent reflections
2452 reflections with *I* > 2σ(*I*)
*R*_int_ = 0.021

#### Refinement

*R*[*F* ^2^ > 2σ(*F* ^2^)] = 0.030
*wR*(*F* ^2^) = 0.080
*S* = 1.04
2543 reflections
351 parameters
1 restraint
H atoms treated by a mixture of independent and constrained refinement
Δρ_max_ = 0.17 e Å^−3^
Δρ_min_ = −0.16 e Å^−3^

### 

Data collection: *APEX2* (Bruker, 2005 ▶); cell refinement: *SAINT* (Bruker, 2005 ▶); data reduction: *SAINT*; program(s) used to solve structure: *SHELXS97* (Sheldrick, 2008 ▶); program(s) used to refine structure: *SHELXL97* (Sheldrick, 2008 ▶); molecular graphics: *PLATON* (Spek, 2009 ▶); software used to prepare material for publication: *publCIF* (Westrip, 2010 ▶).

## Supplementary Material

Crystal structure: contains datablock(s) I, global. DOI: 10.1107/S1600536811024500/vm2102sup1.cif

Structure factors: contains datablock(s) I. DOI: 10.1107/S1600536811024500/vm2102Isup2.hkl

Supplementary material file. DOI: 10.1107/S1600536811024500/vm2102Isup3.cml

Additional supplementary materials:  crystallographic information; 3D view; checkCIF report

## Figures and Tables

**Table 1:** Hydrogen-bond geometry (Å, °)

*D*—H⋯*A*	*D*—H	H⋯*A*	*D*⋯*A*	*D*—H⋯*A*
N10—H10*N*⋯O11^i^	0.85 (3)	1.98 (3)	2.821 (2)	169 (3)
N20—H20*N*⋯O22^ii^	0.84 (3)	2.15 (3)	2.980 (2)	169 (2)

Symmetry codes: (i) 
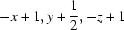
; (ii) 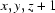.

**Fig. 1.**
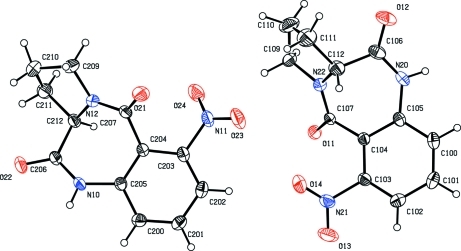
Molecular view of the title compound showing the atom-labeling scheme. Displacement ellipsoids are drawn at the 30% probability level. H atoms are represented as small spheres of arbitrary radii.

**Fig. 2.**
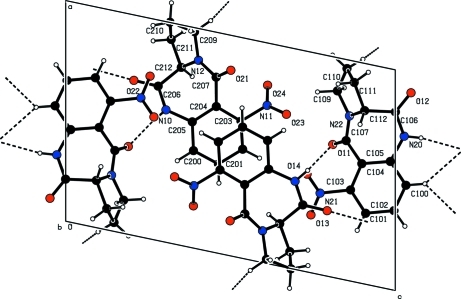
Partial packing view showing the chain formed by N—H···O hydrogen bonds. H atoms not involved in hydrogen bonds have been omitted for clarity.

## supplementary crystallographic information

### Comment

Benzodiazepines form a well known and widely applied class of biologically active compounds (Da Settimo *et al.*, 2007) and are representatives of the family of privileged structures (Herpin *et al.,* 2000). In the area of molecular recognition considerable efforts have been devoted to the synthesis of pyrrolo[2,1 c][1,4]benzodiazepines (PBDs) that can recognize and bind to specific sequences of DNA. They are potential regulators of gene expression with possible application as therapeutic agents in the treatment of genetic disorders including cancer. Furthermore, they can be used as affinity-cleavage reagents in molecular biology (Dervan, 1986). The PBD ring system is also found in natural antitumor antibiotics from Streptomyces species such as Anthramycin (Leimgruber *et al.*, 1975), Tomaymycine (Arima *et al.*, 1983).

The compound, C_24_H_22_N_6_O_8_, crystallizes with two reasonably similar molecules in the asymmetric unit (Fig. 1, r.m.s. deviation of 0.1051 Å for 19 non-H atoms fitted). The nitro and benzene systems are inclined at dihedral angles of 30.0 (3) and 41.0 (3)° in the first and second molecule of the asymetric unit, respectively. The seven-membered diazepine ring adopts a boat conformation (with the two phenylene C atoms representing the stern and the methine C atom the prow). The five-membered pyrrolo ring, which has an envelope conformation, makes dihedral angles of 60.47 (10)° and 54.69 (9)° with the benzene ring of the benzodiazepine in the two independent molecules of the unit.

In the crystal structure, intermolecular N—H···O hydrogen bonds (Fig. 2, Table 1) and π–π stacking interactions (centroid-centroid distance = 3.8023 (7) Å to 3.8946 (7) Å) lead to the formation of a three-dimensional framework.

### Experimental

5-Nitro-1*H*-benzo[*d*][1,3]oxazine-2,4-dione (0.5 g, 2.5 mmol) and *L*-proline (0.29 g, 2.5 mmol) were dissolved in DMF (10 ml) and were then heated under reflux for 3 h. After cooling, the solvent was removed under reduced pressure to yield an oily residue; the residue was then purified over silica gel column chromatography using a mixture of hexane and ethyl acetate (3:1) as eluent. Under these conditions the compound was obtained as colourless crystals.

### Refinement

The H atoms bound to C were treated as riding with their parent atoms [C—H distances are 0.93Å for CH groups with *U*_iso_(H) = 1.2 *U*_eq_(C), and 0.97 Å for CH3 groups with *U*_iso_(H) = 1.5 *U*_eq_(C)]. The nitrogen-bound H atoms were located in a difference Fourier map, and were refined with distance restraints of N–H 0.88±0.01. 2133 Friedel pairs were merged.

### Crystal data

**Table:**

C_12_H_11_N_3_O_4_	*F*(000) = 544
*M**_r_* = 261.24	*D*_x_ = 1.462 Mg m^−^^3^
Monoclinic, *P*2_1_	Mo *K*α radiation, λ = 0.71073 Å
Hall symbol: P 2yb	Cell parameters from 246 reflections
*a* = 10.7364 (2) Å	θ = 2.4–26.3°
*b* = 6.8925 (1) Å	µ = 0.11 mm^−^^1^
*c* = 16.3901 (3) Å	*T* = 296 K
β = 101.870 (1)°	Prism, colourless
*V* = 1186.94 (4) Å^3^	0.23 × 0.20 × 0.15 mm
*Z* = 4	

### Data collection

**Table:**

Bruker APEXII CCD detector diffractometer	2452 reflections with *I* > 2σ(*I*)
Radiation source: fine-focus sealed tube	*R*_int_ = 0.021
graphite	θ_max_ = 26.0°, θ_min_ = 2.5°
ω and φ scans	*h* = −13→13
14768 measured reflections	*k* = −8→8
2543 independent reflections	*l* = −20→20

### Refinement

**Table:**

Refinement on *F*^2^	Primary atom site location: structure-invariant direct methods
Least-squares matrix: full	Secondary atom site location: difference Fourier map
*R*[*F*^2^ > 2σ(*F*^2^)] = 0.030	Hydrogen site location: inferred from neighbouring sites
*wR*(*F*^2^) = 0.080	H atoms treated by a mixture of independent and constrained refinement
*S* = 1.04	*w* = 1/[σ^2^(*F*_o_^2^) + (0.0518*P*)^2^ + 0.1616*P*] where *P* = (*F*_o_^2^ + 2*F*_c_^2^)/3
2543 reflections	(Δ/σ)_max_ < 0.001
351 parameters	Δρ_max_ = 0.17 e Å^−^^3^
1 restraint	Δρ_min_ = −0.16 e Å^−^^3^

### Special details

**Table:**

Geometry. All esds (except the esd in the dihedral angle between two l.s. planes) are estimated using the full covariance matrix. The cell esds are taken into account individually in the estimation of esds in distances, angles and torsion angles; correlations between esds in cell parameters are only used when they are defined by crystal symmetry. An approximate (isotropic) treatment of cell esds is used for estimating esds involving l.s. planes.
Refinement. Refinement of F^2^ against ALL reflections. The weighted R-factor wR and goodness of fit S are based on F^2^, conventional R-factors R are based on F, with F set to zero for negative F^2^. The threshold expression of F^2^ > 2sigma(F^2^) is used only for calculating R-factors(gt) etc. and is not relevant to the choice of reflections for refinement. R-factors based on F^2^ are statistically about twice as large as those based on F, and R- factors based on ALL data will be even larger.

### Fractional atomic coordinates and isotropic or equivalent isotropic displacement parameters (Å^2^)

**Table:**

	*x*	*y*	*z*	*U*_iso_*/*U*_eq_	
C100	0.4871 (2)	0.4617 (3)	1.03240 (11)	0.0410 (4)	
C101	0.3608 (2)	0.4649 (3)	0.99613 (13)	0.0445 (5)	
C102	0.32175 (19)	0.4505 (3)	0.91009 (14)	0.0442 (5)	
C103	0.41314 (17)	0.4215 (3)	0.86410 (11)	0.0359 (4)	
C104	0.54297 (16)	0.4047 (3)	0.89854 (10)	0.0311 (4)	
C105	0.58012 (18)	0.4374 (3)	0.98486 (10)	0.0336 (4)	
C106	0.8095 (2)	0.5177 (3)	1.00041 (13)	0.0483 (5)	
C107	0.63255 (16)	0.3127 (3)	0.85085 (10)	0.0316 (4)	
C109	0.84963 (18)	0.3029 (4)	0.82441 (13)	0.0479 (5)	
C110	0.9600 (2)	0.4396 (6)	0.8535 (2)	0.0749 (9)	
C111	0.9040 (3)	0.6286 (5)	0.8754 (2)	0.0752 (9)	
C112	0.7860 (2)	0.5679 (4)	0.90711 (14)	0.0485 (5)	
C200	0.44163 (18)	0.4931 (3)	0.38760 (12)	0.0399 (4)	
C201	0.40473 (19)	0.4968 (3)	0.46329 (13)	0.0440 (5)	
C202	0.4948 (2)	0.5030 (3)	0.53689 (12)	0.0430 (4)	
C203	0.62060 (19)	0.5134 (3)	0.53182 (11)	0.0374 (4)	
C204	0.66333 (16)	0.5194 (3)	0.45691 (10)	0.0318 (4)	
C205	0.57090 (16)	0.4986 (3)	0.38404 (10)	0.0318 (4)	
C206	0.70022 (18)	0.4042 (3)	0.28050 (11)	0.0378 (4)	
C207	0.79608 (17)	0.5876 (3)	0.45641 (11)	0.0360 (4)	
C209	0.98134 (19)	0.5664 (4)	0.39103 (15)	0.0522 (6)	
C210	1.0011 (2)	0.4484 (5)	0.31649 (15)	0.0655 (8)	
C211	0.9243 (2)	0.2650 (4)	0.32000 (15)	0.0559 (6)	
C212	0.80720 (17)	0.3350 (3)	0.35062 (11)	0.0369 (4)	
H100	0.5118	0.4760	1.0899	0.049*	
H101	0.3004	0.4767	1.0292	0.053*	
H102	0.2362	0.4603	0.8845	0.053*	
H10A	0.8258	0.2995	0.7640	0.058*	
H10B	0.8700	0.1724	0.8451	0.058*	
H10N	0.539 (3)	0.528 (5)	0.2657 (18)	0.056 (8)*	
H112	0.7192	0.6665	0.8932	0.058*	
H11A	0.8815	0.7115	0.8267	0.090*	
H11B	0.9636	0.6972	0.9182	0.090*	
H11C	1.0174	0.3867	0.9019	0.090*	
H11D	1.0070	0.4595	0.8096	0.090*	
H200	0.3803	0.4870	0.3385	0.048*	
H201	0.3187	0.4950	0.4649	0.053*	
H202	0.4709	0.5002	0.5883	0.052*	
H20A	1.0469	0.5388	0.4399	0.063*	
H20B	0.9812	0.7043	0.3793	0.063*	
H20N	0.716 (2)	0.416 (5)	1.0780 (17)	0.058 (7)*	
H212	0.7765	0.2341	0.3837	0.044*	
H21A	0.9722	0.1718	0.3584	0.067*	
H21B	0.9005	0.2058	0.2653	0.067*	
H21C	0.9703	0.5183	0.2649	0.079*	
H21D	1.0905	0.4183	0.3208	0.079*	
N10	0.60011 (15)	0.4905 (3)	0.30409 (9)	0.0369 (4)	
N11	0.71691 (17)	0.5079 (3)	0.61116 (9)	0.0464 (4)	
N12	0.85585 (14)	0.5013 (3)	0.40290 (9)	0.0371 (4)	
N20	0.70780 (16)	0.4399 (3)	1.02707 (10)	0.0413 (4)	
N21	0.36959 (16)	0.4213 (3)	0.77185 (11)	0.0479 (4)	
N22	0.74763 (15)	0.3889 (3)	0.86106 (10)	0.0380 (4)	
O11	0.60185 (13)	0.1634 (2)	0.81012 (8)	0.0435 (4)	
O12	0.91105 (16)	0.5401 (4)	1.04775 (12)	0.0739 (6)	
O13	0.26234 (16)	0.3669 (4)	0.74408 (12)	0.0761 (6)	
O14	0.44340 (18)	0.4853 (4)	0.73230 (10)	0.0743 (6)	
O21	0.84147 (15)	0.7225 (3)	0.50119 (10)	0.0571 (4)	
O22	0.70331 (15)	0.3858 (3)	0.20681 (8)	0.0581 (5)	
O23	0.6951 (2)	0.5986 (4)	0.66967 (9)	0.0723 (6)	
O24	0.80876 (17)	0.4033 (4)	0.61259 (10)	0.0725 (6)	

### Atomic displacement parameters (Å^2^)

**Table:**

	*U*^11^	*U*^22^	*U*^33^	*U*^12^	*U*^13^	*U*^23^
C100	0.0594 (12)	0.0344 (10)	0.0313 (8)	0.0026 (9)	0.0144 (8)	−0.0011 (8)
C101	0.0534 (12)	0.0395 (11)	0.0477 (11)	0.0024 (9)	0.0268 (9)	−0.0014 (9)
C102	0.0350 (9)	0.0462 (12)	0.0522 (11)	0.0019 (9)	0.0107 (8)	−0.0011 (10)
C103	0.0352 (9)	0.0384 (10)	0.0328 (8)	0.0023 (8)	0.0039 (7)	0.0010 (8)
C104	0.0335 (8)	0.0321 (9)	0.0275 (7)	−0.0022 (7)	0.0057 (6)	0.0010 (7)
C105	0.0420 (9)	0.0288 (9)	0.0290 (8)	0.0006 (8)	0.0054 (7)	−0.0002 (7)
C106	0.0483 (11)	0.0426 (11)	0.0478 (11)	−0.0081 (10)	−0.0043 (9)	−0.0095 (10)
C107	0.0311 (8)	0.0397 (10)	0.0225 (7)	−0.0023 (8)	0.0021 (6)	0.0011 (7)
C109	0.0339 (10)	0.0676 (15)	0.0441 (10)	0.0059 (10)	0.0124 (8)	0.0114 (11)
C110	0.0398 (12)	0.097 (2)	0.0907 (19)	−0.0091 (14)	0.0187 (12)	0.021 (2)
C111	0.0662 (17)	0.079 (2)	0.0801 (18)	−0.0344 (16)	0.0148 (14)	0.0114 (17)
C112	0.0483 (11)	0.0458 (12)	0.0499 (11)	−0.0153 (10)	0.0064 (9)	0.0005 (10)
C200	0.0364 (9)	0.0400 (10)	0.0417 (9)	−0.0023 (9)	0.0046 (7)	0.0022 (9)
C201	0.0381 (10)	0.0452 (11)	0.0519 (11)	−0.0033 (9)	0.0163 (8)	0.0039 (10)
C202	0.0537 (11)	0.0398 (10)	0.0407 (9)	0.0029 (10)	0.0217 (8)	0.0050 (9)
C203	0.0479 (10)	0.0353 (9)	0.0289 (8)	0.0061 (9)	0.0079 (7)	0.0014 (8)
C204	0.0358 (9)	0.0313 (9)	0.0282 (8)	0.0027 (7)	0.0059 (7)	0.0000 (7)
C205	0.0359 (9)	0.0294 (8)	0.0299 (8)	0.0005 (8)	0.0065 (7)	0.0018 (7)
C206	0.0412 (9)	0.0434 (10)	0.0273 (8)	−0.0008 (9)	0.0038 (7)	−0.0013 (8)
C207	0.0346 (9)	0.0420 (10)	0.0292 (8)	0.0010 (8)	0.0015 (7)	−0.0022 (8)
C209	0.0338 (10)	0.0653 (15)	0.0589 (12)	−0.0050 (10)	0.0124 (9)	−0.0021 (12)
C210	0.0449 (11)	0.100 (2)	0.0558 (13)	0.0031 (14)	0.0205 (10)	−0.0026 (15)
C211	0.0461 (12)	0.0721 (17)	0.0486 (11)	0.0161 (12)	0.0078 (9)	−0.0136 (12)
C212	0.0393 (10)	0.0408 (10)	0.0298 (8)	0.0036 (8)	0.0047 (7)	−0.0026 (8)
N10	0.0374 (8)	0.0469 (9)	0.0236 (7)	0.0050 (8)	−0.0001 (6)	0.0032 (7)
N11	0.0565 (10)	0.0552 (11)	0.0274 (7)	0.0124 (10)	0.0083 (7)	0.0032 (8)
N12	0.0316 (7)	0.0454 (9)	0.0339 (7)	−0.0014 (7)	0.0054 (6)	−0.0026 (8)
N20	0.0489 (9)	0.0438 (10)	0.0264 (7)	−0.0011 (8)	−0.0033 (6)	−0.0014 (7)
N21	0.0385 (9)	0.0599 (11)	0.0410 (8)	0.0143 (9)	−0.0017 (7)	−0.0011 (9)
N22	0.0335 (7)	0.0454 (9)	0.0350 (7)	−0.0040 (7)	0.0070 (6)	−0.0002 (7)
O11	0.0392 (7)	0.0551 (9)	0.0365 (7)	−0.0099 (7)	0.0085 (5)	−0.0170 (7)
O12	0.0528 (9)	0.0925 (16)	0.0643 (10)	−0.0183 (10)	−0.0158 (8)	−0.0133 (11)
O13	0.0474 (9)	0.1032 (18)	0.0660 (11)	0.0102 (11)	−0.0155 (8)	−0.0227 (12)
O14	0.0711 (11)	0.1173 (18)	0.0343 (7)	0.0125 (13)	0.0102 (7)	0.0148 (11)
O21	0.0456 (8)	0.0651 (11)	0.0592 (9)	−0.0130 (8)	0.0075 (7)	−0.0283 (9)
O22	0.0600 (9)	0.0874 (13)	0.0262 (6)	0.0125 (10)	0.0070 (6)	−0.0042 (8)
O23	0.0948 (13)	0.0859 (14)	0.0326 (7)	0.0271 (12)	0.0053 (8)	−0.0093 (9)
O24	0.0681 (10)	0.1011 (16)	0.0439 (8)	0.0377 (12)	0.0012 (7)	0.0013 (10)

### Geometric parameters (Å, °)

**Table:**

C100—H100	0.9300	C203—N11	1.486 (2)
C100—C101	1.365 (3)	C203—C202	1.372 (3)
C101—H101	0.9300	C204—C207	1.502 (3)
C101—C102	1.390 (3)	C204—C203	1.397 (2)
C102—H102	0.9300	C204—C205	1.394 (2)
C103—N21	1.488 (2)	C205—N10	1.410 (2)
C103—C102	1.369 (3)	C205—C200	1.402 (3)
C104—C107	1.499 (2)	C206—N10	1.352 (3)
C104—C105	1.407 (2)	C206—O22	1.222 (2)
C104—C103	1.397 (2)	C207—O21	1.222 (3)
C105—N20	1.403 (2)	C209—H20B	0.9700
C105—C100	1.397 (3)	C209—H20A	0.9700
C106—N20	1.366 (3)	C209—C210	1.519 (4)
C106—O12	1.211 (3)	C210—H21D	0.9700
C107—N22	1.321 (2)	C210—H21C	0.9700
C107—O11	1.234 (2)	C211—H21B	0.9700
C109—H10B	0.9700	C211—H21A	0.9700
C109—H10A	0.9700	C211—C210	1.516 (5)
C109—C110	1.512 (4)	C212—H212	0.9800
C110—H11D	0.9700	C212—C211	1.525 (3)
C110—H11C	0.9700	C212—C206	1.525 (3)
C111—H11B	0.9700	N10—H10N	0.85 (3)
C111—H11A	0.9700	N11—O24	1.218 (3)
C111—C110	1.508 (5)	N11—O23	1.207 (2)
C112—H112	0.9800	N12—C209	1.470 (2)
C112—C106	1.537 (3)	N12—C212	1.462 (3)
C112—C111	1.524 (3)	N12—C207	1.329 (3)
C200—H200	0.9300	N20—H20N	0.84 (3)
C200—C201	1.378 (3)	N21—O13	1.208 (3)
C201—H201	0.9300	N21—O14	1.206 (3)
C202—H202	0.9300	N22—C109	1.477 (3)
C202—C201	1.383 (3)	N22—C112	1.461 (3)
			
C105—C100—H100	119.4	C204—C203—N11	118.34 (17)
C101—C100—H100	119.4	C202—C203—N11	117.53 (17)
C101—C100—C105	121.28 (18)	C202—C203—C204	124.06 (18)
C102—C101—H101	119.8	C203—C204—C207	119.82 (16)
C100—C101—H101	119.8	C205—C204—C207	122.44 (15)
C100—C101—C102	120.41 (17)	C205—C204—C203	116.44 (16)
C101—C102—H102	121.0	C200—C205—N10	116.68 (16)
C103—C102—H102	121.0	C204—C205—N10	123.03 (15)
C103—C102—C101	117.92 (18)	C204—C205—C200	120.24 (16)
C104—C103—N21	119.29 (16)	N10—C206—C212	116.22 (15)
C102—C103—N21	116.65 (17)	O22—C206—C212	122.90 (18)
C102—C103—C104	123.93 (17)	O22—C206—N10	120.88 (18)
C105—C104—C107	120.83 (16)	N12—C207—C204	116.80 (17)
C103—C104—C107	121.21 (15)	O21—C207—C204	119.76 (17)
C103—C104—C105	116.48 (16)	O21—C207—N12	123.36 (18)
N20—C105—C104	122.95 (17)	H20A—C209—H20B	109.1
C100—C105—C104	119.53 (18)	C210—C209—H20B	111.1
C100—C105—N20	117.48 (16)	N12—C209—H20B	111.1
N20—C106—C112	115.31 (17)	C210—C209—H20A	111.1
O12—C106—C112	123.4 (2)	N12—C209—H20A	111.1
O12—C106—N20	121.2 (2)	N12—C209—C210	103.11 (19)
N22—C107—C104	116.70 (17)	H21C—C210—H21D	108.9
O11—C107—C104	120.41 (16)	C209—C210—H21D	110.9
O11—C107—N22	122.60 (18)	C211—C210—H21D	110.9
H10A—C109—H10B	109.1	C209—C210—H21C	110.9
C110—C109—H10B	111.2	C211—C210—H21C	110.9
N22—C109—H10B	111.2	C211—C210—C209	104.39 (19)
C110—C109—H10A	111.2	H21A—C211—H21B	109.0
N22—C109—H10A	111.2	C212—C211—H21B	111.0
N22—C109—C110	102.7 (2)	C210—C211—H21B	111.0
H11C—C110—H11D	108.6	C212—C211—H21A	111.0
C109—C110—H11D	110.4	C210—C211—H21A	111.0
C111—C110—H11D	110.4	C210—C211—C212	103.9 (2)
C109—C110—H11C	110.4	C211—C212—H212	110.6
C111—C110—H11C	110.4	C206—C212—H212	110.6
C111—C110—C109	106.8 (2)	N12—C212—H212	110.6
H11A—C111—H11B	109.0	C206—C212—C211	113.29 (16)
C112—C111—H11B	110.9	N12—C212—C211	102.79 (17)
C110—C111—H11B	110.9	N12—C212—C206	108.64 (17)
C112—C111—H11A	110.9	C205—N10—H10N	113.2 (18)
C110—C111—H11A	110.9	C206—N10—H10N	117.2 (18)
C110—C111—C112	104.1 (2)	C206—N10—C205	128.68 (16)
C106—C112—H112	110.5	O24—N11—C203	116.55 (17)
C111—C112—H112	110.5	O23—N11—C203	117.90 (18)
N22—C112—H112	110.5	O23—N11—O24	125.44 (18)
C111—C112—C106	115.0 (2)	C212—N12—C209	112.85 (16)
N22—C112—C106	107.16 (18)	C207—N12—C209	122.05 (19)
N22—C112—C111	102.8 (2)	C207—N12—C212	125.10 (16)
C205—C200—H200	119.8	C105—N20—H20N	112.6 (18)
C201—C200—H200	119.8	C106—N20—H20N	117.8 (19)
C201—C200—C205	120.49 (18)	C106—N20—C105	127.69 (17)
C202—C201—H201	119.8	O13—N21—C103	117.34 (19)
C200—C201—H201	119.8	O14—N21—C103	116.24 (18)
C200—C201—C202	120.46 (18)	O14—N21—O13	126.3 (2)
C201—C202—H202	121.0	C112—N22—C109	113.29 (17)
C203—C202—H202	121.0	C107—N22—C109	122.77 (19)
C203—C202—C201	118.00 (18)	C107—N22—C112	123.91 (18)

### Hydrogen-bond geometry (Å, °)

**Table:**

*D*—H···*A*	*D*—H	H···*A*	*D*···*A*	*D*—H···*A*
N10—H10N···O11^i^	0.85 (3)	1.98 (3)	2.821 (2)	169 (3)
N20—H20N···O22^ii^	0.84 (3)	2.15 (3)	2.980 (2)	169 (2)

Symmetry codes: (i) −*x*+1, *y*+1/2, −*z*+1; (ii) *x*, *y*, *z*+1.
